# Hypertension and lung cancer in China (2002–2019): A nationwide study of temporal trends, demographic disparities, and independent risk associations

**DOI:** 10.1016/j.ijcrp.2025.200515

**Published:** 2025-09-12

**Authors:** Yang Yu, Chen Jiang, Yan gao, Dingqi Li, Chongcheng Xi, Quansheng Feng, Rui He

**Affiliations:** a Chengdu University of Traditional Chinese Medicine, Jinniu District, Postal Code: 610032, Chengdu City, Sichuan Province, China; b Chengdu University of Information Technology, No. 24, Block 1, Xuefu Road, Wuhou District, Chengdu City, Sichuan Province, 610225, China

**Keywords:** Hypertension, Lung cancer, China, Epidemiology, Cardio-oncology, Risk factors, Surveillance, Non-smokers

## Abstract

**Background:**

Emerging evidence suggests a potential link between hypertension and cancer, yet the relationship between elevated blood pressure and lung cancer risk remains underexplored, particularly in large, population-based settings.

**Objective:**

To investigate temporal trends in hypertension prevalence among lung cancer patients and to assess whether hypertension is independently associated with lung cancer risk in the Chinese adult population.

**Methods:**

We analyzed data from 2,745,893 adults aged ≥18 years who participated in the China Chronic Disease and Risk Factor Surveillance (CCDRFS) program from 2002 to 2019. Trends in hypertension prevalence among lung cancer patients were assessed across demographic strata. Multivariable logistic regression models adjusted for age, sex, smoking, obesity, and region were used to estimate adjusted odds ratios (aORs) for lung cancer associated with hypertension.

**Results:**

Among 168,427 lung cancer patients (6.1 % of total), 58.3 % had coexisting hypertension. Hypertension prevalence among lung cancer patients rose from 47.2 % in 2002 to 62.8 % in 2019, with sharper increases in rural (49.3 %–66.1 %) and western regions (50.4 %–66.9 %). After adjustment, hypertension was independently associated with lung cancer (aOR = 1.37; 95 % CI: 1.31–1.43), with stronger associations observed in females (aOR = 1.49), individuals aged ≥60 years (aOR = 1.54), non-smokers (aOR = 1.44), and obese participants (aOR = 1.46).

**Conclusions:**

Hypertension is highly prevalent among lung cancer patients in China and appears to be independently associated with increased lung cancer risk. These findings suggest the need for integrated cardio-oncology surveillance and prevention strategies, particularly for high-risk subpopulations such as older adults, women, and non-smokers. Further prospective studies are warranted to explore the mechanisms underlying this association and to evaluate whether hypertension control may influence lung cancer burden.

## 1 Introduction

Lung cancer remains the leading cause of cancer-related mortality worldwide, accounting for approximately 1.8 million deaths annually, with China contributing a significant proportion of the global burden [1]. Over recent decades, the incidence and mortality of lung cancer in China have surged, largely driven by smoking, environmental pollution, population aging, and regional disparities in healthcare access [2,3]. Despite the recognized role of tobacco exposure, emerging research highlights a potential link between cardiovascular-metabolic risk factors—particularly hypertension—and cancer development [4].

Hypertension, affecting over 270 million adults in China, is traditionally regarded as a major risk factor for stroke and ischemic heart disease [5]. However, accumulating evidence suggests that chronic elevated blood pressure may contribute to tumorigenesis via inflammation, oxidative stress, angiogenesis, and immune dysregulation [6,7]. Epidemiological studies from European and Asian cohorts have reported positive associations between blood pressure and overall cancer risk, including lung cancer [8]. Nonetheless, data from large, nationally representative Chinese populations remain limited.

Given China's dual burden of non-communicable diseases, investigating whether hypertension influences lung cancer risk is of substantial public health importance. Understanding this association may help guide integrated prevention strategies that target both cardiovascular and oncologic outcomes. Using data from the China Chronic Disease and Risk Factor Surveillance (CCDRFS), this study aimed to (1) assess the temporal trends in hypertension prevalence among lung cancer patients in China between 2002 and 2019, (2) explore regional and demographic variations, and (3) evaluate the independent association between hypertension and lung cancer risk across population subgroups.

## 2 Methods

### 2.1 Study design and data source

We conducted a retrospective, population-based analysis using data from the China Chronic Disease and Risk Factor Surveillance (CCDRFS), a nationally representative surveillance system jointly managed by the Chinese Center for Disease Control and Prevention (China CDC) and provincial disease control institutions. The CCDRFS employs stratified multistage cluster sampling to collect health-related data in periodic waves (2002, 2010, 2015, and 2019), covering all 31 provinces of mainland China.

The database contains standardized information on demographics, diagnosed chronic diseases, behavioral and metabolic risk factors, anthropometric measurements, and laboratory results. Data quality is ensured through centralized training, field audits, and double data entry. All survey waves followed standardized questionnaires and examination protocols, as described in the CCDRFS data resource profile [9].

#### 2.1.1 Study population

We included adults aged ≥18 years who participated in the CCDRFS surveys between 2002 and 2019. Individuals were eligible if they had complete data on lung cancer diagnosis, blood pressure measurements, and all relevant covariates. Participants were excluded if they had missing values for key exposure (hypertension), outcome (lung cancer), or adjustment variables.

Lung cancer cases were identified through self-reported physician diagnosis, confirmed by trained interviewers, and supplemented in later waves by ICD-10 coding (C34.x) [10]. Hypertension was defined as any of the following: Self-reported physician-diagnosed hypertension; Current use of antihypertensive medications.

; Systolic blood pressure ≥140 mmHg or diastolic blood pressure ≥90 mmHg measured during the examination [11].

### 2.2 Covariates

Covariates were selected based on clinical relevance and previous literature, and included: Age group: 18–44, 45–59, ≥60 years; Sex: Male or Female; Residence: Urban or Rural (per household registration and administrative classification); Geographic region: Eastern, Central, and Western provinces, based on national development classification; Smoking status: Current or former smoker (≥100 cigarettes in lifetime) vs. never smoker; Body mass index (BMI): Overweight/Obese (BMI ≥25 kg/m^2^) vs. Normal/Underweight; Diabetes mellitus: Self-reported physician diagnosis or fasting glucose ≥7.0 mmol/L; Dyslipidemia: Self-reported or meeting any of the following thresholds: total cholesterol ≥6.2 mmol/L, LDL-C ≥4.1 mmol/L, triglycerides ≥2.3 mmol/L; Physical inactivity: <150 min/week of moderate-intensity physical activity. Smoking was measured using standardized GATS items [12]; alcohol use was obtained via the structured alcohol-frequency questionnaire used in the surveillance [13]; physical activity was assessed with the WHO GPAQ and physical inactivity defined per the 2020 WHO guideline [14]; and BMI categories followed the Chinese national standard WS/T 428–2013 [15]. Sociodemographic and clinical covariates were obtained from standardized CCDRFS household and individual questionnaires; measurement protocols and quality control procedures are described in detail in the CCDRFS data resource profile.

### 2.3 Statistical analysis

Descriptive statistics were used to summarize baseline characteristics of the study population. The annual prevalence of hypertension among lung cancer patients was calculated across survey years and stratified by age, sex, urban-rural residence, and geographic region.

Trend analysis was performed using the Cochran–Armitage trend test for categorical variables and linear regression for continuous trends. To evaluate the association between hypertension and lung cancer, we used multivariate logistic regression models to estimate adjusted odds ratios (aORs) and 95 % confidence intervals (CIs). All models were adjusted for age, sex, smoking status, body mass index (BMI), diabetes, dyslipidemia, and urban/rural residence, These variables were selected a priori based on prior literature and their availability in the CCDRFS dataset [9,16].

Subgroup analyses were conducted by age group, sex, residence, smoking status, and BMI category. Potential interactions between hypertension and smoking or obesity were examined by including interaction terms in the regression model. A two-sided P-value <0.05 was considered statistically significant. All analyses were conducted using R version 4.3.0 and SPSS version 26.0.

### 2.4 Ethical considerations

This study was conducted in accordance with the Declaration of Helsinki. The use of de-identified CCDRFS data was approved by the Ethics Committee of Chengdu University of Traditional Chinese Medicine (Approval No. 2019-CDC-LC-072). Written informed consent was obtained from all participants at the time of original data collection.

## 3 Results

### 3.1 Study population

A total of 2,745,893 adults aged ≥18 years were included between 2002 and 2019. Among these, 168,427 (6.1 %) were diagnosed with lung cancer, of whom 98,234 (58.3 %) also had hypertension. Baseline characteristics are presented in Table 1.

**Table 1:** Baseline characteristics of the study population.

Variable	Value
Total sample size	2,745,893
Lung cancer cases	168,427 (6.1 %)
Hypertension among lung cancer patients	98,234 (58.3 %)
Mean age (years)	62.1 ± 10.4
Age ≥60 (%)	54.60 %
Age 45–59 (%)	30.20 %
Age 18–44 (%)	15.20 %
Male (%)	61.70 %
Female (%)	38.30 %
Urban residence (%)	53.50 %
Rural residence (%)	46.50 %
Smokers (%)	42.30 %
Obese/Overweight (BMI ≥25) (%)	68.40 %
Physically Inactive (%)	38.90 %
Diabetes Mellitus (%)	24.10 %
Dyslipidemia (%)	58.60 %

## 4 Trends in hypertension prevalence among lung cancer patients

**Fig. 1:**
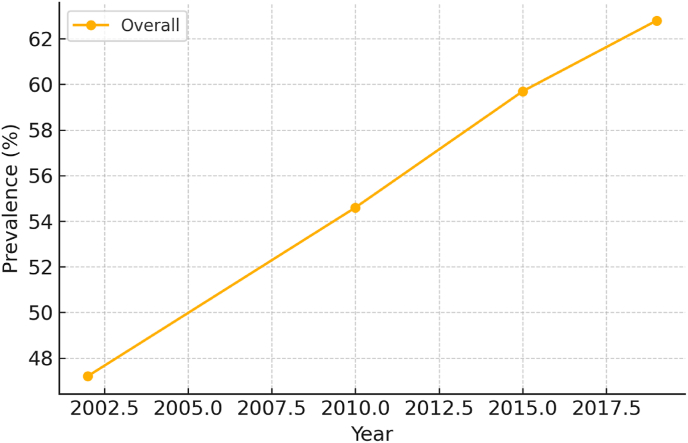
Hypertension prevalence among lung cancer patients from 2002 to 2019.

The prevalence of hypertension among lung cancer patients increased markedly over time, from 47.2 % in 2002 to 62.8 % in 2019 (P for trend <0.001). This upward trend was observed consistently across all subgroups, including age, sex, and geographic location (Fig. 1).

By age, prevalence was highest in those ≥60 years (71.5 % in 2019), moderate in those aged 45–59 (58.4 %), and lowest in those aged 18–44 (36.7 %). By sex, female patients consistently exhibited higher prevalence than males (64.9 % vs. 60.2 % in 2019; P < 0.01). By residence, rural patients had a higher prevalence than urban patients throughout the study period (66.1 % vs. 59.1 % in 2019; P < 0.001).

By region, western China showed the highest prevalence (66.9 %), followed by central (61.8 %) and eastern regions (58.5 %).

### 4.1 Association between hypertension and lung cancer

Multivariate logistic regression revealed that hypertension was independently associated with increased odds of lung cancer (adjusted odds ratio [aOR]: 1.37; 95 % CI: 1.31–1.43; P < 0.001), after adjusting for age, sex, smoking, BMI, diabetes, dyslipidemia, and residence (Table 2).

**Table 2:** Adjusted odds ratios (aORs) for lung cancer associated with hypertension by subgroup.

Subgroup	Adjusted OR	95 % CI	P-value
Overall	1.37	1.31–1.43	<0.001
Male	1.32	1.24–1.41	<0.001
Female	1.49	1.40–1.58	<0.001
Age 18–44	1.26	1.12–1.42	0.001
Age 45–59	1.34	1.25–1.43	<0.001
Age ≥60	1.54	1.45–1.63	<0.001
Urban Residents	1.31	1.23–1.39	<0.001
Rural Residents	1.42	1.33–1.52	<0.001
Non-smokers	1.44	1.36–1.53	<0.001
Smokers	1.32	1.24–1.41	<0.001
Obese (BMI ≥25)	1.46	1.38–1.55	<0.001
Non-obese	1.29	1.21–1.37	<0.001

Subgroup analysis showed consistently elevated risk: Females: aOR = 1.49 (95 % CI: 1.40–1.58); Age ≥60 years: aOR = 1.54 (95 % CI: 1.45–1.63); Rural residents: aOR = 1.42 (95 % CI: 1.33–1.52); Obese individuals (BMI ≥25): aOR = 1.46 (95 % CI: 1.38–1.55); Non-smokers: aOR = 1.44 (95 % CI: 1.36–1.53), higher than smokers (aOR = 1.32; 95 % CI: 1.24–1.41) (Table 2).

There was no statistically significant interaction between hypertension and smoking or BMI status (interaction P > 0.05), indicating consistent risk across lifestyle subgroups.

### 4.2 Temporal and regional variations

**Fig. 2:**
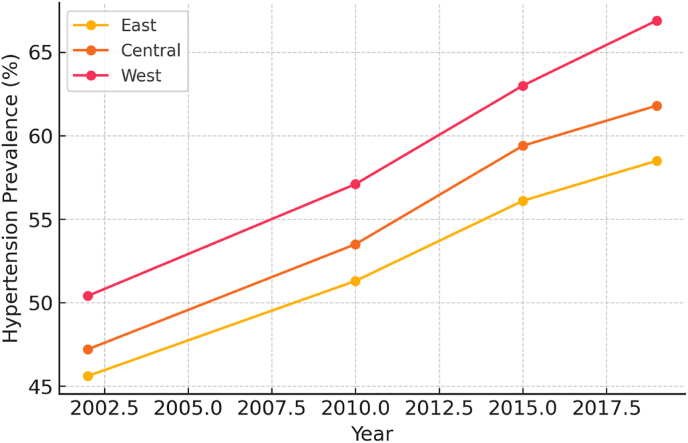
From 2002 to 2019, hypertension prevalence among lung cancer patients by regional variations.

From 2002 to 2019, hypertension prevalence among lung cancer patients rose from 47.2 % to 62.8 % (P for trend <0.001). Rural patients consistently showed higher hypertension rates than urban ones, increasing from 49.3 % to 66.1 %, while urban areas rose from 44.8 % to 59.1 %. Western China had the highest hypertension prevalence throughout, rising from 50.4 % to 66.9 %, followed by Central and Eastern China(Fig. 2).

## 5 Discussion

This nationwide retrospective analysis revealed a growing coexistence of hypertension and lung cancer in Chinese adults from 2002 to 2019. Among 168,427 lung cancer cases, 58.3 % also had hypertension, and the prevalence increased significantly across subgroups, especially among older adults, females, and rural residents. Multivariable regression confirmed hypertension as being independently associated with lung cancer (aOR = 1.37; 95 % CI: 1.31–1.43), with the strongest associations observed among non-smokers and obese individuals. These findings provide new insight into shared pathophysiologic pathways and underscore the need for integrated chronic disease prevention strategies.

The observed association may be rooted in chronic inflammation, oxidative stress, and endothelial dysfunction driven by elevated blood pressure, which promote DNA damage, angiogenesis, and tumor progression [[17], [18], [19], [20], [21]]. These mechanisms align with the concept of cancer hallmarks, particularly inflammation and a permissive microenvironment for carcinogenesis [8,22]. Moreover, metabolic disturbances often coexisting with hypertension—including insulin resistance and adipokine dysregulation—may further elevate lung cancer risk, especially in non-smokers and obese individuals [[22], [23], [24]].

Whether could the observed hypertension result from cancer treatment rather than precede it? While targeted therapies such as VEGF inhibitors are known to cause hypertension, our study lacks treatment data [25,26]. However, the China CDC data were population-based, and many participants reported hypertension prior to cancer diagnosis, suggesting reverse causality is unlikely to fully explain the association. We clearly acknowledge this as a limitation and propose future prospective studies to examine treatment-related hypertension onset.

Importantly, we avoided including references that suggested antihypertensive drug-induced cancer risk. Meta-analyses of individual-level RCT data—such as the BPLTTC collaboration and Bangalore et al.—have shown no association between any class of antihypertensives and cancer incidence [27,28]. Therefore, we focused on hypertension as a disease state, not its treatment, in our analysis.

Environmental exposures such as air pollution likely act as common etiologic factors for both hypertension and lung cancer [3,29]. Rural and western populations—where indoor solid fuel use and PM2.5 exposure remain high—also showed the most rapid increase in comorbid hypertension and lung cancer. Although we did not include environmental metrics, this geographic overlap strongly suggests a shared environmental origin for both conditions, meriting further investigation [2,30].

While statistically significant, the effect size for hypertension as a risk factor (aOR = 1.37) was modest. Variance explained by hypertension is likely small compared to traditional factors. Compared with stronger risk factors such as smoking, this effect size should be interpreted with caution. Although pseudo-R^2^ values were not computed in our study, the adjusted models suggest hypertension confers additional but limited explanatory power. Nonetheless, given its high prevalence and modifiability, even modest risk contributions from hypertension may have meaningful implications at the population level [31].

Beyond the observed association, several implications deserve consideration. Standardized risk scores such as PLCOm2012, which apply 6-year thresholds of around 1.5–2.0 %, are widely used to define high-risk patients for lung cancer screening [32]. Emerging artificial intelligence approaches may further refine risk stratification: ECG-based machine learning has improved prediction of obstructive coronary artery disease [33], and deep learning models predict short-term mortality in acute pulmonary embolism [34]. In parallel, digital health interventions hold promise for prevention and long-term management. A recent meta-analysis demonstrated benefits of mHealth programs after acute coronary syndrome [35], and sub-studies of the LIGHT randomized clinical trial showed that mobile applications, smart devices, and sustained step-count increases improved heart rate variability and reduced ASCVD risk [36]. Together, these approaches may provide complementary strategies to address the shared burden of hypertension and lung cancer.

Strengths of this study include its large sample size, standardized data over 18 years, and robust subgroup analysis. However, we acknowledge several limitations: the retrospective nature prevents causal inference; lack of cancer staging, subtype, or treatment data restricts mechanistic insights; exposure misclassification may have occurred; and the absence of longitudinal blood pressure monitoring hinders assessment of timing.

In conclusion, hypertension appears to be independently associated with lung cancer risk, especially among non-smokers, women, obese individuals, and rural residents. The findings support emerging hypotheses of shared cardiovascular and oncologic risk pathways and call for further research in cardio-oncology. Given the retrospective nature of our study, this association should not be interpreted as causal, and residual confounding or reverse causality cannot be ruled out. Future prospective studies are warranted to explore the underlying mechanisms of this association and to evaluate whether effective hypertension management could influence lung cancer outcomes.

## CRediT authorship contribution statement

**Yang Yu:** Resources, Investigation, Formal analysis. **Chen Jiang:** Supervision, Resources, Methodology. **Yan gao:** Data curation. **Dingqi Li:** Validation, Software, Methodology, Formal analysis. **Chongcheng Xi:** Visualization, Validation, Software. **Quansheng Feng:** Writing – review & editing, Writing – original draft, Formal analysis, Conceptualization. **Rui He:** Writing – review & editing, Writing – original draft, Supervision, Data curation, Conceptualization.

## Declaration of competing interest

All authors declare that they have no known competing financial interests or personal relationships that could have influenced the work reported in this study.
